# Modelling the Surface Plasmon Spectra of an ITO Nanoribbon Grating Adjacent to a Liquid Crystal Layer

**DOI:** 10.3390/ma13071523

**Published:** 2020-03-26

**Authors:** Victor Yu. Reshetnyak, Victor I. Zadorozhnii, Igor P. Pinkevych, Timothy J. Bunning, Dean R. Evans

**Affiliations:** 1 Physics Faculty, Taras Shevchenko National University of Kyiv, 01601 Kyiv, Ukraine; VReshetnyak@univ.kiev.ua (V.Y.R.); v.i.zador@gmail.com (V.I.Z.); 2 Air Force Research Laboratory, Materials and Manufacturing Directorate, Wright-Patterson Air Force Base, OH 45433, USA

**Keywords:** surface plasmon, plasmon spectra, nanoribbon grating, ITO, liquid crystal

## Abstract

The reflection and transmission coefficients of an indium tin oxide (ITO) nanoribbon grating placed between a nematic liquid crystal (LC) layer and an isotropic dielectric medium are calculated in the infrared region. Reflection and transmission spectra in the range of 1–5 μm related to the surface plasmon excitation in the ITO nanoribbons are obtained. Dependence of the peak spectral position on the grating spacing, the ribbon aspect ratio, and the 2D electron concentration in the nanoribbons is studied. It is shown that director reorientation in the LC layer influences the plasmon spectra of the grating, enabling a control of both the reflection and transmission of the system. The data obtained with our model are compared to the results obtained using COMSOL software, giving the similar results.

## 1. Introduction

Surface plasmons (SPs) can be excited in the gratings comprising metallic or semiconductor ribbons by an electromagnetic wave with a wavelength much larger than the ribbon size. Initially, SPs in the grating structures were excited in graphene micro- and nanoribbon gratings. In the infrared (IR) region, resonance peaks in reflection, transmission, and absorption spectra related to the SP excitation in these gratings were observed. By adjusting the ribbon width, grating spacing, or electron gas concentration, it is possible to control the SP resonance frequency, and therefore, change the optical properties of the grating-based devices [1,2,3,4,5,6,7,8].

Indium tin oxide (ITO) belongs to the group of transparent conductive oxides, which have reduced electric losses and provide tunable optical properties [9,10,11,12,13,14]. Thin films of ITO are widely used as transparent conductive coatings for making various devices, in particular, liquid crystal displays, antireflective coatings in solar cells, window electrodes in optoelectronic devices, etc. [15,16,17,18,19,20,21]. Important for applications, the combination of electrical and optical properties stimulates researchers to investigate the ITO properties and its plasmonic spectra. In recent years, transparent conductive oxides including ITO are considered as an alternative to noble metals in different plasmonic device applications [22,23,24,25,26]. In this paper, we theoretically study the influence of parameters of the ITO nanoribbon grating on its reflection, and transmission spectra in the IR region, *λ* = 1–5 μm, where the efficient excitation of SPs was observed in the thin ITO films [18,19,20]. The ITO grating is adjacent to the liquid crystal (LC) layer. The LC is an anisotropic dielectric, and its permittivity can be easily managed reorienting the LC director by external electric or magnetic field. The LCs are known for their applications in biosensing [27,28,29,30]. We suggest that LCs in combination with plasmon nanostructures can also be used for sensory applications. Therefore, we also study an influence of the LC director reorientation in the LC layer on the ITO grating plasmon spectra. The data obtained in the model of the infinitely thin grating are compared with the results obtained using COMSOL software.

The paper is organized as follows: Section 2 introduces a model of the ITO-grating structure with an adjacent LC layer, and presents the basic equations allowing for the calculations of reflection and transmission coefficients of the system. Results of numerical calculations of the coefficients and their discussion are presented in Section 3. In Section 4, some brief conclusions are presented.

## 2. Model of the Grating Structure

**Figure 1:**
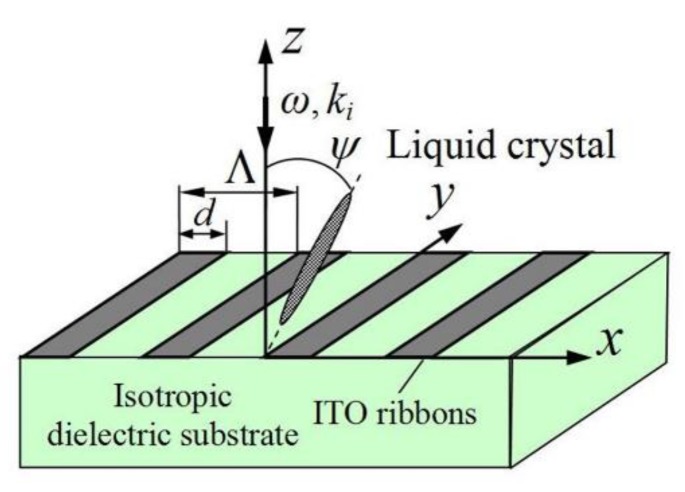
Schematic showing the electromagnetic wave with the wave vector $k_{i}$ incident from the LC layer on the ITO grating located on the isotropic dielectric. Λ is the grating spacing and *d* is the width of the ITO ribbons.

The ITO nanoribbon grating is placed in the *xy*-plane between a nematic LC layer and an isotropic dielectric substrate. The grating ribbons are directed along the *y*-axis, *d* is a width of the ITO ribbon, and Λ is the grating spacing (Figure 1). A plane monochromatic electromagnetic wave is incident along the *z*-axis on the grating from the side of the LC layer and excites the SPs in the ITO nanoribbons.

To simplify calculations, we consider the incident wave to be a transverse magnetic (TM) wave with a magnetic vector $H_{i}$ directed in the LC layer along the *y*-axis. The TM-wave electric vector $E_{i}$ should have a component perpendicular to the grating ribbons to excite the SPs; therefore, for the wave incident on the ITO grating, we write:

(1) $$E_{i}=\left(E_{ix}e_{x}+E_{iz}e_{z}\right)e^{i(k_{i}r-\omega t)},\;H_{i}=H_{i}e_{y}e^{i(k_{i}r-\omega t)},\;k_{i}=(0,0,-k_{i}).$$

The LC permittivity tensor is defined as $\varepsilon_{ij}=n_{o}^{2}\delta_{ij}+(n_{e}^{2}-n_{o}^{2})s_{i}s_{j}$, i=x,y,z, where $s_{i}$ is the LC director component, $n_{o}$ and $n_{e}$ are the refractive indices of the ordinary and extraordinary waves, respectively [31]. The nematic director is defined as:

(2) s=(sinψ,0,cosψ)

where ψ is the director angle with respect to the *z*-axis. For the sake of simplicity, the LC director profile is assumed to be homogenous throughout the whole LC layer, and is restricted only to the case of the director reorientation in the *xz*-plane. Then, as it follows from Maxwell’s equations, $H_{i}=-\left(k_{i}/\omega \mu_{0}\right)E_{ix}$, $E_{iz}=-(\varepsilon_{xz}/\varepsilon_{zz})E_{ix}$, and the wave vector of the incident wave is related to the wave frequency by the dispersion equation $k_{i}=\left(\omega /c\right)\sqrt{\varepsilon_{xx}-\varepsilon_{xz}{}^{2}/\varepsilon_{zz}}$.

We intend to study reflection and transmission of the electromagnetic wave in the ITO grating structure presented in Figure 1, and calculate the corresponding reflection (R) and transmission (T) coefficients defined as:

(3) R=|Re(Er×Hr*)|/|Re(Ei×Hi*)|,T=|Re(Et×Ht*)|/|Re(Ei×Hi*)|,

where $E_{r},H_{r}$ and $E_{t},H_{t}$ are the electric and magnetic vectors of the reflected and transmitted waves, respectively.

In general, it is necessary to solve Maxwell’s equations in the bulk of the grating material satisfying the boundary conditions for electromagnetic field at the boundaries with the LC layer and isotropic dielectric substrate. This is a complex coupled problem; however, in the case where the ITO ribbons’ thickness becomes infinitesimally small, h→0*,* the problem simplifies significantly. In this case, it is not necessary to solve the Maxwell’s equations in the grating bulk, only the boundary conditions relating the electromagnetic fields from both sides of the grating must be satisfied. These boundary conditions in the case of infinitely thin grating (*z = 0*) are as follows:

(4) $$\begin{array}{l} \left[e_{z}\times \left(E_{i}+E_{r}-E_{t}\right)\right]|{}_{z=0}\;e_{y}=0,\\ \left[e_{z}\times \left(H_{i}+H_{r}-H_{t}\right)-j_{s}\right]|{}_{z=0}\;e_{x}=0, \end{array}$$

where $j_{s}$ is an electric current density in the plane of the grating and $e_{x},e_{y},e_{z}$ are the unit vectors along the Cartesian axes.

The wave vectors of the reflected and transmitted waves are in the *xz*-plane. Using the Fourier-Floquet expansion with respect to the coordinate *x*, the electric and magnetic vectors of the reflected and transmitted waves can be written as:

(5) $$\begin{array}{l} E_{r}=\sum_{n}E_{0rn}e^{i(k_{n}x+k_{rn}z-\omega \;t)},H_{r}=\sum_{n}H_{0rn}e^{i(k_{n}x+k_{rn}z-\omega \;t)},\\ E_{t}=\sum_{n}E_{0tn}e^{i(k_{n}x+k_{tn}z-\omega \;t)},H_{t}=\sum_{n}H_{0tn}e^{i(k_{n}x+k_{tn}z-\omega \;t)}, \end{array}$$

where $k_{n}=2\pi n/\Lambda$ and *n* is the number of the Fourier-Floquet spatial harmonic.

Substituting $E_{t}$, $H_{t}$ from Equation (5) into the Maxwell equations in the LC and $E_{t}$, $H_{t}$ into the Maxwell equations in the isotropic dielectric material with permittivity ε, we obtain [7]:

(6) $$\begin{array}{l} E_{r}=\frac{c/\omega }{\varepsilon_{zz}\sqrt{\left(\varepsilon_{xx}-\varepsilon_{xz}{}^{2}/\varepsilon_{zz}\right)}}\sum_{n}\left[\left(\varepsilon_{zz}k_{rn}+\varepsilon_{xz}k_{n}\right)e_{x}-\left(\varepsilon_{xz}k_{rn}+\varepsilon_{xx}k_{n}\right)e_{z}\right]a_{n}e^{i(k_{n}x+k_{rn}z-\omega \;t)},\\ H_{r}=\varepsilon_{0}c\sqrt{\left(\varepsilon_{xx}-\varepsilon_{xz}{}^{2}/\varepsilon_{zz}\right)}\sum_{n}a_{n}e^{i(k_{n}x+k_{rn}z-\omega \;t)}e_{y}, \end{array}$$

where $k_{rn}=\sqrt{\left(\omega^{2}/c^{2}-k_{n}{}^{2}/\varepsilon_{zz}\right)\left(\varepsilon_{xx}-\varepsilon_{xz}{}^{2}/\varepsilon_{zz}\right)}-\left(\varepsilon_{xz}/\varepsilon_{zz}\right)k_{n}$ and

(7) $$\begin{array}{l} E_{t}=-\frac{c}{\omega \sqrt{\varepsilon }}\sum_{n}\left(k_{tn}e_{x}+k_{n}e_{z}\right)b_{n}e^{i(k_{n}x-k_{tn}z-\omega \;t)},\\ H_{t}=\varepsilon_{0}c\sqrt{\varepsilon }\sum_{n}b_{n}e^{i(k_{n}x-k_{tn}z-\omega \;t)}e_{y}, \end{array}$$

where $k_{tn}=\sqrt{{\left(\omega /c\right)}^{2}\varepsilon -k_{n}{}^{2}}$. In Equations (6) and (7), $a_{n}$ and $b_{n}$ are the coefficients of the Fourier-Floquet expansions for the reflected and transmitted waves, respectively.

The current density in the plane of the grating can be written as:

(8) $$j_{s}=\sigma (x)E_{tx}(z=0)e_{x}$$

where σ(x)=σ within the ITO nanoribbons (nΛ<x<nΛ+d) and σ(x)=0 between the nanoribbons (nΛ+d<x<(n+1)Λ); σ is the ITO nanoribbon surface conductivity.

Substituting Equations (1), (6), and (7) into the first boundary condition (Equation (4)), we obtain an expression for the coefficients $b_{n}$ in terms of the coefficients $a_{n}$:

(9) $$b_{n}=-\frac{1}{A_{n}^{\mathrm{is}}\left(\omega \right)}\left[\frac{\omega }{c}E_{ix}\delta_{n0}+A_{n}^{\mathrm{LC}}\left(\omega \right)a_{n}\right]$$

where we introduced the following notations:

(10) $$A_{n}^{\mathrm{LC}}\left(\omega \right)=\sqrt{\frac{\omega^{2}}{c^{2}}-\frac{4\pi^{2}n^{2}}{\varepsilon_{zz}\Lambda^{2}}},\;A_{n}^{\mathrm{is}}\left(\omega \right)=\sqrt{\frac{\omega^{2}}{c^{2}}-\frac{4\pi^{2}n^{2}}{\varepsilon \Lambda^{2}}}$$

After substituting Equations (1), (6), and (7) into the second boundary condition (Equation (4)), and performing some straightforward algebra, we arrive at the following system of equations for the coefficients $a_{n}$:

(11) $$\begin{array}{l} \left[A_{n}^{\mathrm{LC}}\left(\omega \right)\left(\frac{\sigma d}{\varepsilon_{0}\omega \Lambda }+\frac{\sqrt{\varepsilon }}{A_{n}^{\mathrm{is}}\left(\omega \right)}\right)+\sqrt{\varepsilon_{xx}-\frac{\varepsilon_{xz}{}^{2}}{\varepsilon_{zz}}}\right]a_{n}+\frac{i\sigma }{2\pi \omega \varepsilon_{0}}\sum_{m(m\neq n)}\frac{\left(e^{\frac{2i\pi (m-n)d}{\Lambda }}-1\right)A_{m}^{\mathrm{LC}}\left(\omega \right)}{\left(n-m\right)}a_{m}\\ =\left[\sqrt{\varepsilon_{xx}-\frac{\varepsilon_{xz}{}^{2}}{\varepsilon_{zz}}}-\frac{\sigma d}{\varepsilon_{0}c}-\frac{\sqrt{\varepsilon }\omega /c}{A_{n}^{\mathrm{is}}\left(\omega \right)}\right]E_{ix}\delta_{n0}+\frac{i\sigma \left(1-e^{\frac{-2i\pi nd}{\Lambda }}\right)}{2\pi \varepsilon_{0}nc}E_{ix}(1-\delta_{n0}), \end{array}$$

Equations (9) and (11) were obtained in our previous papers related to the plasmon resonances in the graphene and MoS_2_ microribbon gratings [7,32].

Considering the case where the ribbon thickness becomes infinitely thin, h→0, the ITO as a monolayer film can be treated like graphene with σ representing the ITO ribbon surface conductivity. For its calculations, we use the Drude form for 2D conductivity [33]:

(12) $$\sigma =\frac{n_{2D}e^{2}\tau }{m_{e}^{*}}\frac{1+i\omega \tau }{1+{\left(\omega \tau \right)}^{2}}$$

where $n_{2D}$ is the 2D electron concentration, τ is the electron relaxation time, and $m_{e}^{*}$ is the electron effective mass.

Substituting Equation (12) into Equation (11), one can numerically solve them and calculate the coefficients $a_{n}$, and then, using Equation (9), the coefficients $b_{n}$. Now from Equations (6) and (7), we can determine the electric and magnetic vectors of the reflected and transmitted waves, and then finally, calculate the reflection and transmission coefficients defined by Equation (3).

For numerical calculations, as an example of the LC layer adjacent to the ITO grating, we used the material parameters for the nematic LC W1791 as it possesses great optical anisotropy with $n_{o}\approx 1.53$ and $n_{e}\approx 1.94$ at *λ* = 1.064 μm [34]; we set the bottom isotropic substrate to be a glass with a permittivity of ε= 1.52^2^. The electron effective mass in the ITO film is taken to be $m_{e}^{*}=0.37m_{e}$, where $m_{e}$ is the mass of electron [21]. The free electron concentration $n_{2D}$ and electron relaxation time τ depend to a considerable extent on the ITO sample preparation, and in this study parameters, $n_{2D}$ and τ are considered as variables.

## 3. Results and Discussion

**Figure 2:**
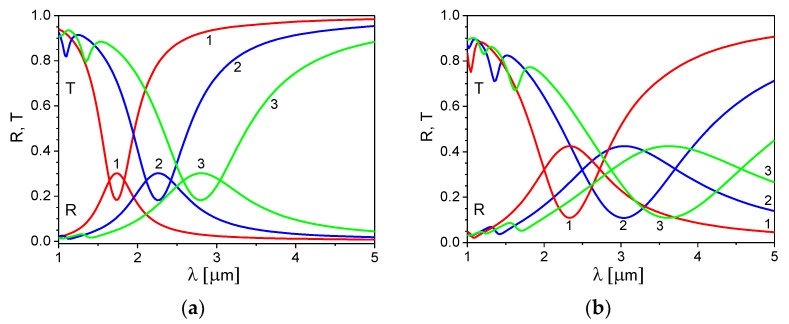
Reflection and transmission spectra of the ITO nanoribbon grating for different values of the grating spacing Λ: 300 nm—curves 1, 500 nm—curves 2, and 750 nm—curves 3. The ribbon aspect ratio: (**a**) d/Λ = 0.5 and (**b**) d/Λ = 0.75. τ = 6.7fs [35], $n_{2D}$ = 5 × 10^19^ m^−2^, and the director angle ψ = π/2.

In Figure 2a,b, we present the reflection and transmission spectra of the ITO nanoribbon grating for several values of the grating spacing Λ and fixed values of the ribbon width to grating spacing ratio, d/Λ = 0.5 (Figure 2a) and d/Λ = 0.75 (Figure 2b). In both cases, the director orientation is planar (ψ = π/2). The grating spacing values were chosen to obtain a plasmonic spectral response in the IR region, *λ* = 1–5 μm.

**Figure 3:**
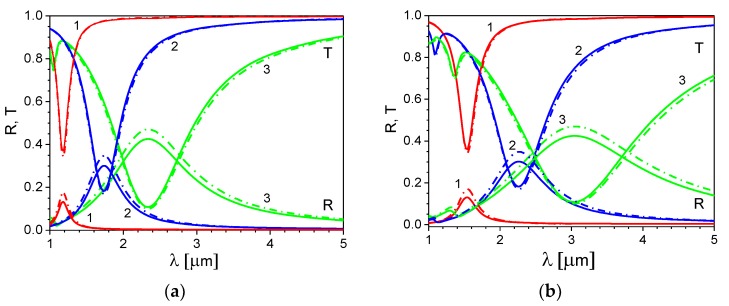
Reflection and transmission spectra of the ITO nanoribbon grating for different values of the ribbon aspect ratio d/Λ and two orientations of the nematic director. Grating spacing: (**a**) Λ = 300 nm and (**b**) Λ = 500 nm; d/Λ = 0.25—curves 1, d/Λ = 0.5—curves 2, and d/Λ = 0.75—curves 3; planar director orientation (ψ = π/2)—solid lines and homeotropic director orientation (ψ = 0)—dotted-dashed lines. τ = 6.7fs and $n_{2D}$ = 5 × 10^19^ m^−2^.

Peaks in the reflection and transmission spectra observed in Figure 2a,b are the results of the excitation of the SP mode in the ITO nanoribbons. These peaks shift to the long wavelengths with an increase of the grating spacing or the ribbon aspect ratio d/Λ. A magnitude of the peaks and their width increase with an increase of the ratio d/Λ. This can be seen more clearly in Figure 3a,b, which shows the reflection and transmission coefficients calculated for different values of the ribbon width to grating spacing ratio, d/Λ= 0.25, 0.5, and 0.75, for fixed values of the grating spacing, Λ = 300 nm (Figure 3a) and Λ = 500 nm (Figure 3b). In these figures, we show the reflection and transmission coefficients for two cases of the LC director orientation: the director in the planar state (ψ = π/2) and the director in the homeotropic state (ψ = 0).

As seen in Figure 3a,b, the wavelength of the plasmon resonance and the magnitude of the corresponding peak increase with an increase of the ribbon aspect ratio d/Λ. Therefore, one can control the ITO nanoribbon grating spectra by changing the value of the grating spacing Λ or the ribbon aspect ratio d/Λ. However, by varying Λ, only the wavelength of the plasmon resonance peak changes, whereas by varying the ratio d/Λ, the magnitude of the peak also changes.

From Figure 3a,b, it is also seen that reorientation of the LC director strongly influences the magnitude of the reflection peak; the same tendency was also observed in the SP spectra of the graphene grating [7]. In particular, reorientation of the LC director from the planar (ψ = π/2) to homeotropic (ψ = 0) state leads to an increase of the reflection peak magnitude by approximately 30% for d/Λ = 0.25, 15% for d/Λ = 0.5, and 10% for d/Λ = 0.75. We would like to note that in the current study, we show only a principal opportunity of the plasmonic spectra tuning with help of LCs. For real engineering application, the LC parameters (e.g., birefringence) and the plasmonic structure parameters should be optimized, but this is beyond the scope of the current study.

**Figure 4:**
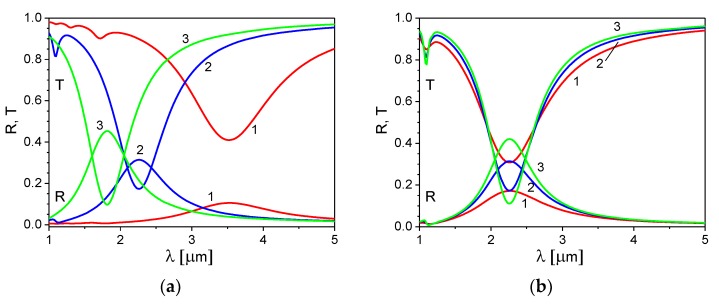
Influence of the 2D electron concentration (**a**) and the electron relaxation time (**b**) on the reflection and transmission spectra of the ITO nanoribbon grating: (**a**) $n_{2D}$ = 2 × 10^19^ m^−2^—curves 1, 5 × 10^19^ m^−2^—curves 2, 8 × 10^19^ m^−2^—curves 3, and τ = 7 fs; (**b**) τ = 4 fs —curves 1, 7 fs —curves 2, and 10 fs —curves 3; $n_{2D}$ = 5 × 10^19^ m^−2^; and grating spacing Λ = 500 nm, aspect ratio d/Λ = 0.5, and director angle ψ = π/2.

An influence of the 2D electron concentration in the ITO nanoribbons and the electron relaxation time on the reflection and transmission spectra is shown in Figure 4a,b, respectively. The grating spacing and the ribbon aspect ratio are fixed and set equal to Λ = 500 nm and d/Λ= 0.5, respectively; the director orientation is planar.

With an increase of the 2D electron concentration (Figure 4a), the wavelength of the plasmon resonance peak shifts into the short-wavelength side that correlates with a corresponding increase of the plasmon frequency, increase of a magnitude of the resonance peak, and decrease of their width. An increase of the electron relaxation time leads to an increase of the reflection coefficient, and thus a decrease of the transmission coefficient (Figure 4b).

**Figure 5:**
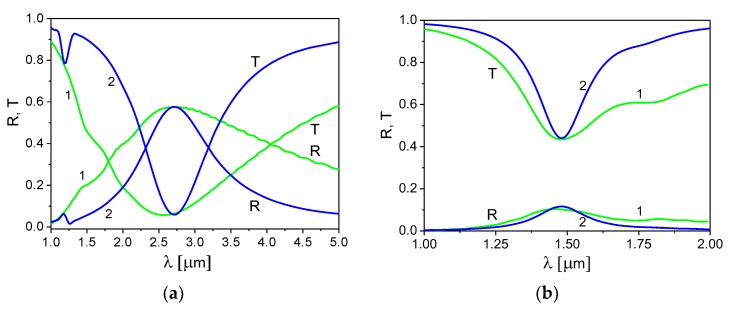
Reflection and transmission spectra of the ITO nanoribbon grating with a grating spacing Λ = 300 nm and a ribbon aspect ratio (**a**) d/Λ = 0.25 and (**b**) d/Λ = 0.75 calculated using COMSOL software for an ITO grating with a thickness h = 120 nm (curves 1) and using an approximation of the infinitely thin grating (curves 2). Parameters used for the approximation of the infinitely thin grating: (**a**) τ = 8 fs and $n_{2D}$ = 3.1 × 10^19^ m^−2^ and (**b**) τ = 13.1 fs and $n_{2D}$ = 3.5 × 10^19^ m^−2^.

In Figure 5, results from calculating reflection and transmission spectra of the ITO nanoribbon grating, using an approximation of infinitely thin gratings (h→0), are compared to those obtained using COMSOL software (COMSOL Multiphysics®5.5, COMSOL Co., Ltd., Shanghai, China) for an ITO grating with a thickness h = 120 nm and dielectric parameters obtained for close ITO thicknesses [36]. As an example, we used a grating spacing of Λ = 300 nm and an aspect ratio d/Λ = 0.25 (Figure 5a) and d/Λ = 0.75 (Figure 5b); the director orientation in the LC layer is homeotropic (ψ = 0).

We were able to get the best agreement between the results for the infinitely thin grating (h→0) and the results of the COMSOL calculations (h = 120 nm) by choosing the 2D electron concentration from the range of (3.1–3.5) × 10^19^ m^−2^ and the electron relaxation time from the range of 8–13.1 fs instead of τ = 5.6 fs used in the COMSOL calculations. Results of the fit indicated that the spectral position and magnitude of the resonance plasmon peaks in the reflection and transmission spectra can be reasonably described in the model of the infinitely thin grating using the 2D electron concentration and electron relaxation time as the fitting parameters although the peaks are narrower.

**Figure 6:**
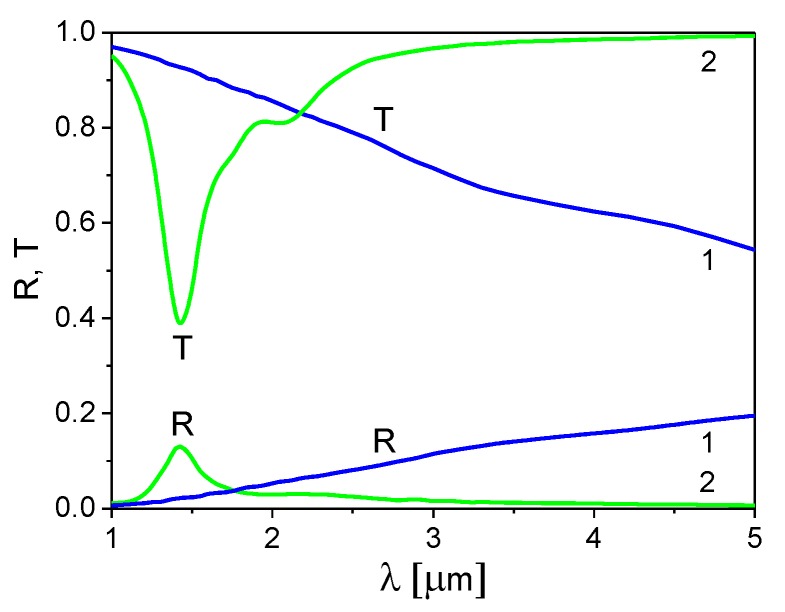
Influence of the director reorientation from the twisted to homeotropic state on the reflection and transmission spectra of the ITO nanoribbon grating: twisted state—curves 1 and homeotropic state—curves 2. Grating spacing Λ = 150 nm and ribbon aspect ratio d/Λ = 0.25. Calculations were performed using COMSOL software.

Finally, it should be noted that it may be possible to control the SPs excitation in the grating by reorienting the director in the LC layer from the twisted to homeotropic state. In the twisted state, the director is oriented along the *x*-axis at the LC layer entrance (see Figure 1) and along the *y*-axis near the ITO grating. It is proposed that the so-called Mauguin regime holds [37,38] such that the polarization of the light wave propagating through the LC layer rotates following the LC director rotation. In this case, the initially *x*-polarized incident light wave gains a polarization parallel to the ITO ribbons near the grating, and therefore cannot excite the SPs. By applying a weak external voltage, one can reorient the director in the LC layer from a twisted to a homeotropic state with the director along the *z*-axis. Here, the incident light wave polarization is not affected in the LC layer. In this case, the light wave propagates to the ITO ribbons with a polarization perpendicular to the grating ribbons and excites the SPs. Using COMSOL software for calculations, in Figure 6, we demonstrate the change in the transmission and reflection of the system when the LC director is reoriented from the twisted orientational state (curves 1) to the homeotropic state (curves 2).

## 4. Conclusions

The reflection and transmission coefficients of an ITO nanoribbon grating placed between a nematic LC and an isotropic dielectric are calculated in the infrared region (1–5 μm). Using the ITO grating with a grating spacing from the range of 300–750 nm, peaks in the reflection and transmission spectra are obtained, which are the result of exciting SPs in ITO nanoribbons. The wavelength of the plasmon resonances in the reflection and transmission spectra increases with an increase of the grating spacing and the ribbon aspect ratio d/Λ, and decreases with an increase of the 2D electron concentration. A magnitude of the resonance peaks in the spectral region 1–5 μm does not change with a grating spacing change, but increases with an increase of the 2D electron concentration, the ratio d/Λ, and the electron relaxation time.

We show that the orientation state of the LC layer placed at the top of the ITO grating influences the magnitude of the plasmon peak in the reflection spectrum. As a result, reorientation of the LC director from the planar to homeotropic state enables the control of the reflection peak within 10–30% of its magnitude depending on the ribbon aspect ratio. The reorientation of the LC director from the twisted to homeotropic state allows the switching ON/OFF of the SPs excitation. Data obtained in the model using the infinitely thin grating approach are compared with results obtained for the ITO grating with thickness of 120 nm using COMSOL software, indicating that this simplified model can describe sufficiently well the spectral position and magnitude of the resonance peaks.
